# Asymmetric Double-Sideband Composite Signal and Dual-Carrier Cooperative Tracking-Based High-Precision Communication–Navigation Convergence Positioning Method

**DOI:** 10.3390/s25113405

**Published:** 2025-05-28

**Authors:** Zhongliang Deng, Zhenke Ding, Xiangchuan Gao, Peijia Liu

**Affiliations:** 1 School of Electrical and Information Engineering, Zhengzhou University, Zhengzhou 450001, China; 2 School of Electronic Engineering, Beijing University of Posts and Telecommunications, Beijing 100876, China

**Keywords:** communication and navigation fusion system, double-sideband signal, 5G, signal combination processing, fuzzy control

## Abstract

To enhance positioning capability and reliability within existing Communication Navigation Fusion Systems (CNFSs), this paper proposes an Asymmetric Double-Sideband Composite Localization Signal (ADCLS) and a dual-carrier aggregation dual-code loop tracking mechanism with fuzzy control. By organically integrating an embedded signal into the original positioning signal, the code loop is optimized via fuzzy control, while the ADCLS signal is processed as an asymmetric double-sideband signal for joint signal extraction. Experimental validation employs the 5G New Radio (NR) Time-Delay Line (TDL) channel model to simulate multipath propagation effects. The results show that this method improves the tracking accuracy of the code loop and the main carrier loop, thereby enhancing the ranging accuracy.

## 1. Introduction

With the expanding coverage of 5G networks, the large-scale deployment of 5G communication infrastructure has created new opportunities for indoor positioning applications [1,2]. While cellular-based positioning technologies (e.g., E911 and smart city systems) offer valuable services, current solutions based on cellular communication often fail to meet the stringent high-precision positioning requirements of industries such as manufacturing, robotics, and autonomous navigation. For instance, cell-ID (CID)-based positioning techniques typically suffer from errors ranging from tens of meters to over a hundred meters [3].

To address these limitations, the Positioning Reference Signal (PRS) in LTE and 5G NR systems has been designed to enhance positioning accuracy [4,5]. However, PRS consumes valuable communication signal bandwidth and restricts the continuous broadcast duration of signals, particularly in dense urban environments [6]. In response, this paper proposes a novel approach where positioning and communication signals are co-transmitted by a Fusion System Base Station (FSBS) within the same frequency band. These signals are differentiated by power levels, with the location signal adopting a Binary Phase Shift Keying (BPSK)-modulated Direct Sequence Spread Spectrum Code Division Multiple Access (DSSS-CDMA) scheme. The receiving terminal estimates the Time Difference of Arrival (TDOA) to calculate precise positioning results [7,8].

The Communication Navigation Fusion System (CNFS) primarily consists of three components: a satellite timing system, a communication–navigation convergence network, and user terminal equipment [9]. Its core architecture involves the indoor deployment of penetration-capable fusion base stations. These base stations synchronize with an external high-precision clock via outdoor satellite timing systems to ensure time and frequency accuracy. Communication and positioning signals are then distributed through wired backhaul links. The terminal estimates positioning by measuring the Time Difference of Arrival (TDOA) of the received signals. The accuracy of TDOA-based ranging primarily depends on the tracking precision of the code loop and the carrier loop. By smoothing the carrier phase pseudo-range based on the tracking performance of both loops, highly accurate distance measurements can be achieved. In practical applications, some systems rely solely on code-based ranging, while others utilize both code and carrier phase information. This paper aims to enhance tracking precision in order to improve overall positioning accuracy.

To address coverage limitations in complex indoor environments (e.g., shadowed zones or multi-floor buildings), supplementary systems are deployed to enhance signal strength while maintaining co-channel integration with cellular networks. This hybrid approach forms a unified communication–navigation network. However, the bandwidth-constrained fusion of positioning and communication signals imposes fundamental limits on positioning accuracy. Addressing this challenge—how to improve positioning accuracy within fixed bandwidth limitations—is critical for advancing CNFS capabilities.

The receiving terminal acquires the communication–navigation fusion signal by generating a pseudocode pair. Leveraging the autocorrelation function’s characteristics, the pseudocode pair achieves maximum autocorrelation peak when synchronized [10,11]. Due to the high code rate of 10.23 MHz, the Partial Match Filter–Fast Fourier Transform (PMF-FFT) method is employed for rapid signal acquisition, yielding coarse pseudocode phase estimates and Doppler frequency shifts [12,13,14].

After acquiring the positioning signal within the communication–navigation integrated system, the terminal enters the tracking phase [15,16]. A multi-channel architecture—typically comprising more than six channels—is established to independently receive signals from multiple CNFS stations [17,18]. Each channel extracts the code phase, carrier phase, and Doppler frequency shift. Positioning is then achieved by solving observation equations and integrating navigation messages [19,20]. However, conventional tracking loops are not compatible with the Asymmetric Double-Sideband Composite Localization Signal (ADCLS) due to its inherent asymmetric double-sideband structure. Therefore, a dedicated tracking loop must be designed to fully exploit the correlation properties of ADCLS.

To achieve more reliable and accurate positioning in constrained environments (e.g., indoors or urban canyons), this paper proposes a fast acquisition method based on the CNFS. The method employs a dual-resident acquisition signal with frequency compensation in the block accumulation domain, enabling high-sensitivity and high-precision frequency acquisition while reducing resource consumption [21]. Borio et al. explored the fusion of pilot and positioning signals, demonstrating that while sharing the same frequency band improves coherent integration time—thereby enhancing acquisition sensitivity—the accuracy of loop tracking remains largely unaffected [22]. Although increasing bandwidth and sampling rates could theoretically improve tracking accuracy, such adjustments are impractical for existing CNFSs due to predefined signal parameters. The concept of meta-signals was first introduced in the literature as a means of treating two signals from different frequency bands as a unified entity to enhance tracking accuracy [23]. However, this approach is limited by the frequency allocations of the original signals and still requires additional spectrum resources. Furthermore, discrepancies in clock sources and the lack of inter-channel correlation impede the realization of optimized joint tracking.

Building upon the existing CNFS, this study aims to improve ranging accuracy and reliability. However, the bandwidth limitations of the deployed hardware make further expansion infeasible. To address this constraint without modifying the system’s overall bandwidth, a narrowband positioning signal is embedded into the original transmission to form an ADCLS. To ensure stable reception of the ADCLS, a dual-carrier, dual-code loop tracking mechanism incorporating fuzzy control is proposed.

The main contributions of this paper are as follows. First, under the constraint of fixed bandwidth in the existing communication–navigation fusion positioning system, an Asymmetric Double-Sideband Composite Localization Signal (ADCLS) is designed to enhance positioning accuracy without occupying additional bandwidth. Second, a dual-carrier, dual-code loop tracking architecture based on fuzzy control is developed to enable stable reception of the asymmetric dual-band signal, thereby fully leveraging the advantages of ADCLS.

The structure of the paper is organized as follows. Section 2 introduces the ADCLS, the fuzzy control-based dual-carrier dual-code tracking loop, and relevant error analysis. Section 3 presents experimental evaluations and performance analysis of the proposed signal and receiver. Section 4 concludes the paper.

## 2. Asymmetric Double-Sideband Composite Location Signal and Reception

### 2.1. Asymmetric Double-Sideband Composite Location Signal

Without modifying the original CNFS or its bandwidth, a narrowband positioning signal is embedded into the existing communication–navigation fusion signal. The two signals are combined in the time domain to form the ADCLS, which is mathematically defined in Equation (1).

(1) $$\begin{array}{c} s\left(t\right)=p_{1}d_{1}\left(t\right)c_{1}\left(t\right)\exp\left[j\left(2\pi f_{1}t+\theta_{1}\right)\right]+p_{2}c_{2}\left(t\right)\exp\left[j\left(2\pi f_{2}t+\theta_{2}\right)\right] \end{array}$$

In Equation (1), $p_{1}$ is the amplitude of the original positioning signal, $d_{1}\left(t\right)$ is the navigation message of the original positioning signal, $c_{1}\left(t\right)$ is the spread spectrum code of the original positioning signal, $f_{1}$ is the carrier frequency of the original positioning signal, $\theta_{1}$ is the carrier phase of the original positioning signal, $p_{2}$ is the amplitude of the embedded positioning signal, $c_{2}\left(t\right)$ is the spread spectrum code of the embedded positioning signal, $f_{2}$ is the carrier frequency of the embedded positioning signal, and $\theta_{2}$ is the carrier phase of the embedded positioning signal. Here, the embedded signal selects Weil code as the spread spectrum code of the positioning signal, whose code length is 1023, code rate is 1.023 MHz, and modulation mode is BPSK. The carrier frequency selection should meet the requirement that the original positioning signal and the embedded positioning signal are orthogonal—that is, Equation (2) is satisfied.

(2) $$\begin{array}{c} \;\int_{0}^{T_{S}}\;c_{1}\left(t\right)\;\exp\left[j\left(\;2\pi \;f_{1}t\;+\;\theta_{1}\right)\right]\times \;\exp\;\left[j\left(2\;\pi \;f_{2}t\;+\;\theta_{2}\;\right)\right]\;dt\;=\;0 \end{array}$$

**Figure 1:**
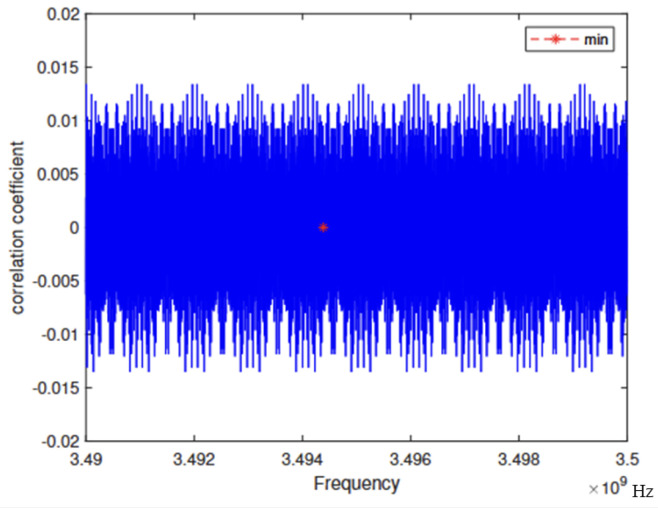
The correlation coefficient.

Let $T_{S}$ denote the coherence integration time. The frequency band of the original positioning signal is set to 3489.77–3510.23 MHz, with a sampling interval of 1000 Hz applied to the lower sideband. As shown in Figure 1, the center frequency of the embedded signal is 3494.39 MHz, where the correlation coefficient reaches a minimum value of 0.000006082. Therefore, the two signals can be considered approximately orthogonal.

Since both the original and embedded positioning signals are generated by the same FSBS, they share a common reference clock and propagation path. As a result, the two signals exhibit strong correlation at the same receiver terminal. Accordingly, Equation (1) can be reformulated as follows:

(3) $$\begin{array}{cc} s(t) & =\left\{p_{1}d_{1}\left(t\right)c_{1}\left(t\right)\exp\left[j\left(2\pi f_{sc}t+\theta_{sc}\right)\right]\right.\left.+p_{2}c_{2}\left(t\right)\exp\left[-j\left(2\pi f_{sc}t+\theta_{sc}\right)\right]\right\}\times \exp\left[j\left(2\pi f_{0}t+\theta_{0}\right)\right]\\ \; & =s_{a}\left(t\right)\times \exp\left(j\left(2\pi f_{0}t+\theta_{0}\right)\right. \end{array}$$

In Equation (3), $f_{0}$ and $\theta_{0}$ are the main carrier frequency and the main carrier phase of the asymmetric double-sideband compound positioning signal, respectively, $f_{sc}$ and $\theta_{sc}$ are the subcarrier frequency and the subcarrier phase of the asymmetric double-sideband compound positioning signal, respectively, where $f_{0}$, $\theta_{0}$, $f_{sc}$, and $\theta_{sc}$ can also be expressed as follows:

(4) $$\begin{array}{cc} f_{sc} & =\left(f_{1}-f_{2}\right)/2\\ \theta_{sc} & =\left(\theta_{1}-\theta_{2}/2\right.\\ f_{0} & =\left(f_{1}+f_{2}\right)/2\\ \theta_{0} & =\left(\theta_{1}+\theta_{2}/2\right. \end{array}$$

$s_{a}\left(t\right)$ can be expressed as

(5) $$\begin{array}{c} s_{a}\left(t\right)=p_{1}d_{1}\left(t\right)c_{1}\left(t\right)\exp\left[j\left(2\pi f_{SC}t+\theta_{SC}\right)\right]+p_{2}c_{2}\left(t\right)\exp\left[-j\left(2\pi f_{SC}t+\theta_{SC}\right)\right] \end{array}$$

Equation (5) can be regarded as a double-sideband signal, but its upper and lower sideband power, pseudo-code code length, pseudo-code code rate, and message information are different. $p_{o}^{2}/p_{e}^{2}=10$ of upper and lower sideband signals. The upper sideband pseudo-code code length is 10230, the code rate is 10.23 MHz, and the carrier frequency is 3500 MHz. The weil code length of the lower band signal is 1023, the code rate is 1.023 MHz, and the carrier frequency is 3494.39 MHz. The modulation mode of the upper and lower sideband is BPSK. Equation (1) can be expressed as

(6) $$\begin{array}{cc} s(t) & =p_{o}d_{o}\left(t\right)c_{o}\left(t\right)\exp\left[j\left(2\pi f_{o}t\;+\;\theta_{o}\right)\right]\;+\;p_{e}c_{e}\left(t\right)\exp\left[j\left(2\pi f_{e}t\;+\;\theta_{e}\right)\right]\\ \; & =\left\{p_{o}d_{o}\left(t\right)c_{o}\left(t\right)\exp\left[j\left(2\pi f_{sc}t+\theta_{sc}\right)\right]\right.\left.+p_{e}c_{e}\left(t\right)\exp\left[-j\left(2\pi f_{sc}t+\theta_{sc}\right)\right]\right\}\times \exp\left[j\left(2\pi f_{0}t+\theta_{0}\right)\right]\\ \; & =\left\{s_{o}\left(t\right)+s_{e}\left(t\right)\right\}\times \exp\left(j\left(2\pi f_{0}t+\theta_{0}\right)\right) \end{array}$$

And $s_{o}\left(t\right)=p_{o}d_{o}\left(t\right)c_{o}\left(t\right)\exp\left[j\left(2\pi f_{sc}t+\theta_{sc}\right)\right]$, $s_{e}\left(t\right)=p_{e}c_{e}\left(t\right)\exp\left[-j\left(2\pi f_{sc}t+\theta_{sc}\right)\right]$, $f_{sc}$ = 2.805 MHz, and $s_{o}\left(t\right)+s_{e}\left(t\right)$ can be regarded as bilateral band signal, and its power spectrum can be expressed as

(7) $$\begin{array}{cc} G_{a}\left(f\right) & =G_{o}\left(f\right)+G_{e}\left(f\right)=p_{o}^{2}G_{o}\left(f-f_{sc}\right)+p_{e}^{2}G_{e}\left(f+f_{sc}\right) \end{array}$$

$G_{o}\left(f\right)$ and $G_{e}\left(f\right)$ in Equation (7), respectively, represent the power spectrum of the original location signal and embedded small location signal, $G_{o}\left(f\right)=T_{c1}{\mathrm{sinc}}^{2}\left(T_{c1}f\right)$, $G_{e}\left(f\right)=T_{c2}{\mathrm{sinc}}^{2}\left(T_{c2}f\right)$, and $T_{c1}$ and $T_{c2}$ are the Weil code lengths of the original bit signal, respectively, that is, $T_{c1}=1/f_{c1}$ and $T_{c2}=1/f_{c2}$, where $f_{c1}$ and $f_{c1}$ are the Weil code rate of the original bit signal and the code rate of the embedded positioning signal, respectively. Then, the power spectrum expression of the composite bilateral band signal can be obtained as follows:

(8) $$\begin{array}{c} G_{a}\left(f\right)=p_{o}^{2}T_{c1}{\mathrm{sinc}}^{2}\left(T_{c1}\left(f-f_{sc}\right)\right)+p_{e}^{2}T_{c2}{\mathrm{sinc}}^{2}\left(T_{c2}\left(f+f_{sc}\right)\right) \end{array}$$

**Figure 2:**
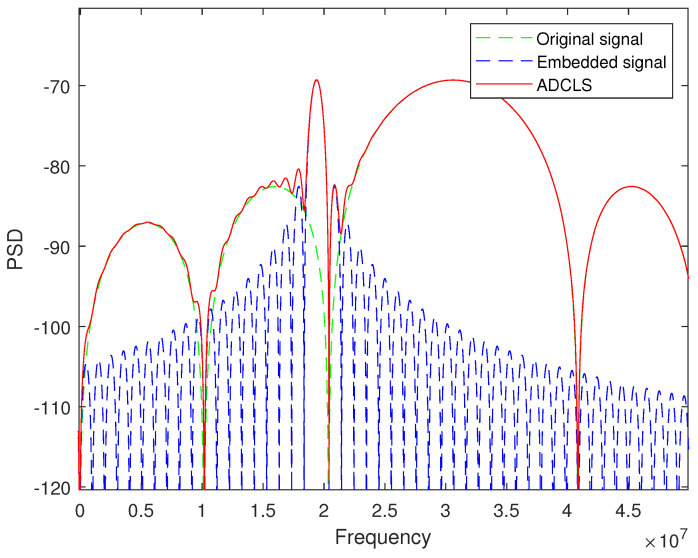
Signal power spectrum.

The power spectrum of the composite asymmetric bilateral band signal of original position signal and embedded small signal is shown in Figure 2.

The autocorrelation function of the ADCLS can be expressed as

(9) $$\begin{array}{cc} R_{A}\left(\tau \right) & =E\left[s_{a}\left(t\right)s_{a}^{*}\left(t-\tau \right)\right]\\ \; & =E\left[\left(s_{o}\left(t\right)+s_{e}\left(t\right)\right){\left(s_{o}\left(t\right)+s_{e}\left(t\right)\right)}^{*}\right]\\ \; & =\left[p_{o}^{2}d_{o}\left(t\right)d_{o}^{∼}\left(t\right)R_{o}\left(\tau \right)\;+\;p_{e}^{2}R_{e}\left(\tau \right)\right]\cos\left(2\pi f_{sc}\tau \right)\\ \; & +j\left[p_{o}^{2}d_{o}\left(t\right)d_{o}^{∼}\left(t\right)R_{o}\left(\tau \right)\;-\;p_{e}^{2}R_{e}\left(\tau \right)\right]\sin\left(2\pi f_{sc}\tau \right) \end{array}$$

**Figure 3:**
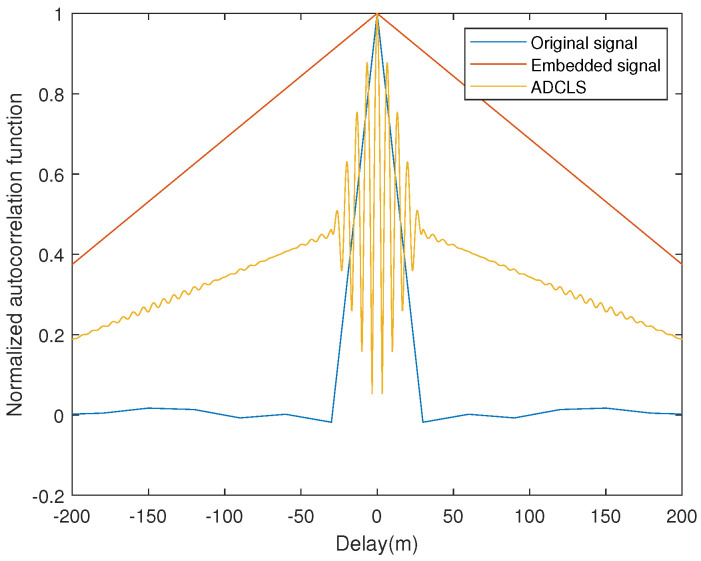
Normalized autocorrelation function.

$d_{o}^{∼}\left(t\right)$ in Equation (9) is the local decoding message, which can be regarded as aligned in normal tracking, namely, $d_{o}\left(t\right)=d_{o}^{∼}\left(t\right)$. At this time, the envelope of correlation function is shown in Figure 3. Figure 3 shows the envelope of its normalized autocorrelation function, whose horizontal coordinate represents the distance corresponding to τ of Equation (9), that is, the distance corresponding to the phase of the delayed code, and τ represents the time delay, but in order to represent its effect on ranging more obviously, it will be converted into a delayed distance based on the pseudo-code rate versus the speed of light. The main peak of the autocorrelation function of ADCLS is sharper, which will inevitably bring better tracking performance. However, because of its multiple peak values, the normal Delay-Locked Loop (DLL) can no longer be used, and the code loop tracking mode should be replaced accordingly.

### 2.2. Asymmetric Double-Carrier Double-Code Loop Combined Tracking Loop Based on Fuzzy Control

**Figure 4:**
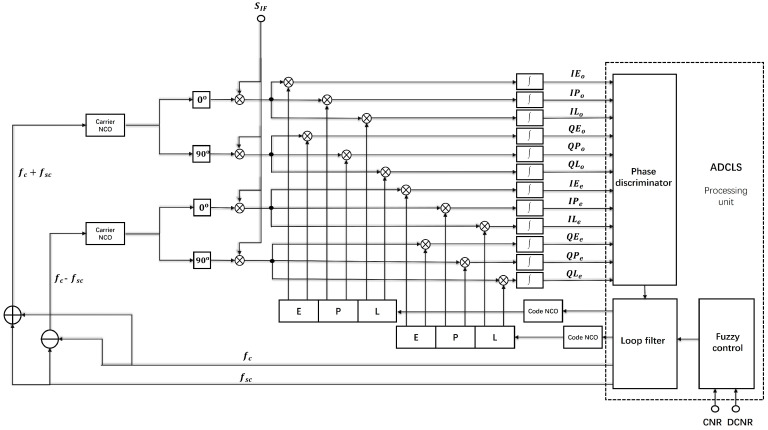
Flow chart of tracking loop algorithm.

The traditional single-channel tracking loop is no longer applicable for the ADCLS. As the ADCLS is a bilateral band signal, existing algorithms such as the Binary Offset Carrier (BOC) Dual Estimation Technique (DET) and the Double Phase Estimator (DPE) can serve as references. However, both DET and DPE are designed for symmetric bilateral band signals and are therefore not directly applicable to the inherently asymmetric nature of the ADCLS. To fully acquire and track the ADCLS while leveraging the inter-channel correlation between its components, this study proposes an asymmetric dual-carrier joint receiving and tracking loop based on fuzzy control. The structure of the proposed algorithm is illustrated in Figure 4.

The ADCLS receiver first separates the input intermediate frequency (IF) signal $S_{IF}$ into its in-phase (I) and quadrature (Q) components. These components are then mixed with both the main and sub-carrier frequencies for carrier removal. After carrier stripping, the resulting signals are correlated with a locally generated Weil code. The local Weil code includes three replicas with different phases: Early (E), Prompt (P), and Late (L), where E corresponds to the early replica, P to the prompt replica, and L to the late replica. Consequently, the coherent integration values for twelve tracking paths can be obtained as follows:

(10) $$\begin{array}{cc} IE_{o}\left(k\right) & \;=\;A_{o}D\left(k\right)T_{coh}R_{o}\left(\tau \;-\;\hat{\tau }\;-\;\frac{d}{2}\right)\frac{\sin\left(\pi \Delta f_{o}T_{coh}\right)}{\pi \Delta f_{o}T_{coh}}\cos\left(\Delta \varphi_{o}\right)\;+\;n_{I}\\ IP_{o}\left(k\right) & \;=\;A_{o}D\left(k\right)T_{coh}R_{o}\left(\tau -\hat{\tau }\right)\frac{\sin\left(\pi \Delta f_{o}T_{coh}\right)}{\pi \Delta f_{o}T_{coh}}\cos\left(\Delta \varphi_{o}\right)+n_{I}\\ IL_{o}\left(k\right) & \;=\;A_{o}D\left(k\right)T_{coh}R_{o}\left(\tau \;-\;\hat{\tau }\;+\;\frac{d}{2}\right)\frac{\sin\left(\pi \Delta f_{o}T_{coh}\right)}{\pi \Delta f_{o}T_{coh}}\cos\left(\Delta \varphi_{o}\right)\;+\;n_{I}\\ QE_{o}\left(k\right) & \;=\;A_{o}D\left(k\right)T_{coh}R_{o}\left(\tau \;-\;\hat{\tau }\;-\;\frac{d}{2}\right)\frac{\sin\left(\pi \Delta f_{o}T_{coh}\right)}{\pi \delta fT_{coh}}\sin\left(\Delta \varphi_{o}\right)\;+\;n_{Q}\\ QP_{o}\left(k\right) & \;=\;A_{o}D\left(k\right)T_{coh}R_{o}\left(\tau \;-\;\hat{\tau }\right)\frac{\sin\left(\pi \Delta f_{o}T_{coh}\right)}{\pi \Delta f_{o}T_{coh}}\sin\left(\Delta \varphi_{o}\right)\;+\;n_{Q}\\ QL_{o}\left(k\right) & \;=\;A_{o}D\left(k\right)T_{coh}R_{o}\left(\tau \;-\;\hat{\tau }\;+\;\frac{d}{2}\right)\frac{\sin\left(\pi \Delta f_{o}T_{coh}\right)}{\pi \Delta f_{o}T_{coh}}\sin\left(\Delta \varphi_{o}\right)\;+\;n_{Q}\\ IE_{e}\left(k\right) & \;=\;A_{e}D\left(k\right)T_{coh}R_{e}\left(\tau \;-\;\hat{\tau }\;-\;\frac{d}{2}\right)\frac{\sin\left(\pi \Delta f_{e}T_{\mathrm{coh}}\right)}{\pi \Delta f_{e}T_{\mathrm{coh}}}\cos\left(\Delta \varphi_{e}\right)\;+\;n_{Q}\\ IP_{e}\left(k\right) & \;=\;A_{e}D\left(k\right)T_{\mathrm{coh}}R_{e}\left(\tau \;-\;\hat{\tau }\right)\frac{\sin\left(\pi \Delta f_{e}T_{\mathrm{coh}}\right)}{\pi \Delta f_{e}T_{\mathrm{coh}}}\cos\left(\Delta \varphi_{e}\right)\;+\;n_{I}\\ IL_{e}\left(k\right) & \;=\;A_{e}D\left(k\right)T_{coh}R_{e}\left(\tau \;-\;\hat{\tau }\;+\;\frac{d}{2}\right)\frac{\sin\left(\pi \Delta f_{e}T_{coh}\right)}{\pi \Delta f_{e}T_{coh}}\cos\left(\Delta \varphi_{e}\right)\;+\;n_{I}\\ QE_{e}\left(k\right) & \;=\;A_{e}D\left(k\right)T_{coh}R_{e}\left(\tau \;-\;\hat{\tau }\;-\;\frac{d}{2}\right)\frac{\sin\left(\pi \Delta f_{e}T_{coh}\right)}{\pi \Delta f_{e}T_{\mathrm{coh}}}\sin\left(\Delta \varphi_{e}\right)\;+\;n_{Q}\\ QP_{e}\left(k\right) & \;=\;A_{e}D\left(k\right)T_{coh}R_{e}\left(\tau \;-\;\hat{\tau }\right)\frac{\sin\left(\pi \Delta f_{e}T_{coh}\right)}{\pi \Delta f_{e}T_{coh}}\sin\left(\Delta \varphi_{e}\right)\;+\;n_{Q}\\ QL_{e}\left(k\right) & \;=\;A_{e}D\left(k\right)T_{\mathrm{coh}}R_{e}\left(\tau \;-\;\hat{\tau }\;+\;\frac{d}{2}\right)\frac{\sin\left(\pi \Delta f_{e}T_{\mathrm{coh}}\right)}{\pi \Delta f_{e}T_{coh}}\sin\left(\Delta \varphi_{e}\right)\;+\;n_{Q}\\ \Delta \varphi_{e} & =\pi \Delta f_{e}T_{co\;h}+\Delta \theta_{e}\\ \Delta \varphi_{o} & =\pi \Delta f_{o}T_{co\hbar }+\Delta \theta_{o}\\ \Delta f_{o} & =f_{c}-{\tilde{f}}_{c}+f_{sc}-\tilde{f_{sc}}\\ \Delta f_{e} & =f_{c}-{\tilde{f}}_{c}-f_{sc}+\tilde{f_{sc}}\\ \Delta \theta_{e} & =\theta_{c}-\tilde{\theta_{c}}-\theta_{sc}+\tilde{\theta_{sc}}\\ \Delta \theta_{o} & =\theta_{c}-{\tilde{\theta }}_{c}+\theta_{sc}-\tilde{\theta_{sc}} \end{array}$$

In Equation (10), $\Delta f_{e}$ and $\Delta f_{o}$ are the carrier frequency errors of the embedded signal and the original location signal, respectively, ${\tilde{f}}_{c}$ and $\tilde{f_{sc}}$ are the local replication main carrier frequency and sub-carrier frequency, $\tilde{\theta_{c}}$ and $\tilde{\theta_{sc}}$ are the local replication main carrier phase and sub-carrier phase, $\Delta \varphi_{e}$ and $\Delta \varphi_{o}$ are the carrier phase errors of the embedded signal and the original location signal and the local replication signal, respectively, $n_{I}$ and $n_{Q}$ represent White Gaussian Noise (WGN) signals with a mean value of 0 and variance of $\delta_{n}^{2}$, *k* represents integral period, and d represents the early and late correlator interval. In this structure, the carrier ring completes carrier identification of FLL and PLL by means of the P branch accumulation system, and the output of the carrier discriminator is the phase error between the received signal and local repeated carrier signal. By using the carrier phase error feedback loop filter and a local Carrier Numerically controlled Oscillator (NCO), the local carrier NCO adjusts the locally generated carrier frequency. The structure of the code loop, through comparing the E and L branch coherent integration values to the position of the peak pseudo-code autocorrelation function, does not depend on the carrier phase and will not be affected by carrier frequency drift. It adjusts the generation rate of the local replication code according to the integral value of E and L to ensure that the local replication code is aligned with the received signal’s Weil code.

The ADCLS processing unit supports both single-frequency and dual-frequency signal tracking modes. During the initial signal acquisition phase, single-frequency processing circuits are employed to independently establish stable tracking for each signal path. This process yields stable estimates of the code phase, carrier phase, and carrier frequency for both signals. Once stable tracking is achieved, the processing unit transitions to a dual-frequency joint tracking mode, enabling improved ranging performance. In scenarios where one of the sidebands experiences significant narrowband interference or signal weakening, a fuzzy control mechanism dynamically adjusts the weighting of each signal path. If interference becomes excessive, the system reduces the affected signal’s weight or disables it entirely, reverting to single-frequency tracking mode. This mode-switching process is entirely handled by software; no modifications to the hardware are required. In the following section, we present the dual-frequency joint tracking algorithm and the fuzzy control strategy in detail.

Signal quality is commonly assessed using the Signal-to-Noise Ratio (SNR), which is defined as the ratio of signal power to noise power. A higher SNR indicates better signal quality. However, since SNR is dependent on the noise bandwidth, it is often unsuitable for direct comparison across systems. To address this, alternative normalized metrics are used. One such metric is the Difference of Carrier-to-Noise Ratio (DCNR), which reflects the contrast in carrier-to-noise values between adjacent signal loads. DCNR is typically used to evaluate signal fluctuations and to identify the presence of interference. In the case of the original baseband signal and the embedded signal, the embedded signal generally occupies a narrower bandwidth and is thus less susceptible to narrowband interference. As a result, it is sufficient to monitor only the Carrier-to-Noise Ratio (CNR) of the original baseband signal to evaluate interference levels.

The fuzzy controller primarily consists of four components: fuzzification, a knowledge base, fuzzy inference, and defuzzification [24,25,26]. In the proposed fuzzy control loop, there are two inputs, CNR and DCNRl, and a single output variable, the weight coefficient. To accurately capture the influence of CNR and DCNR on the weight coefficient, their value ranges are determined through empirical testing and analysis of actual received signals. The observed CNR values range from 20 dB-Hz to 50 dB-Hz, while DCNR varies between −15 and +15. The output of the fuzzy controller, i.e., the weight coefficient, is defined over the range [0, 1]. The corresponding universes of discourse (domains) for the input and output variables are defined as follows.

(11) $$\begin{array}{cc} Y_{1} & =\left\{\begin{array}{ccccc} 20 & 24 & 33 & 41 & 50 \end{array}\right\}\\ Y_{2} & =\left\{\begin{array}{ccccc} 0 & 2 & 5 & 10 & 15 \end{array}\right\}\\ Y_{3} & =\left\{\begin{array}{ccccc} 0 & 0.3 & 0.5 & 0.8 & 1 \end{array}\right\} \end{array}$$

**Figure 5:**
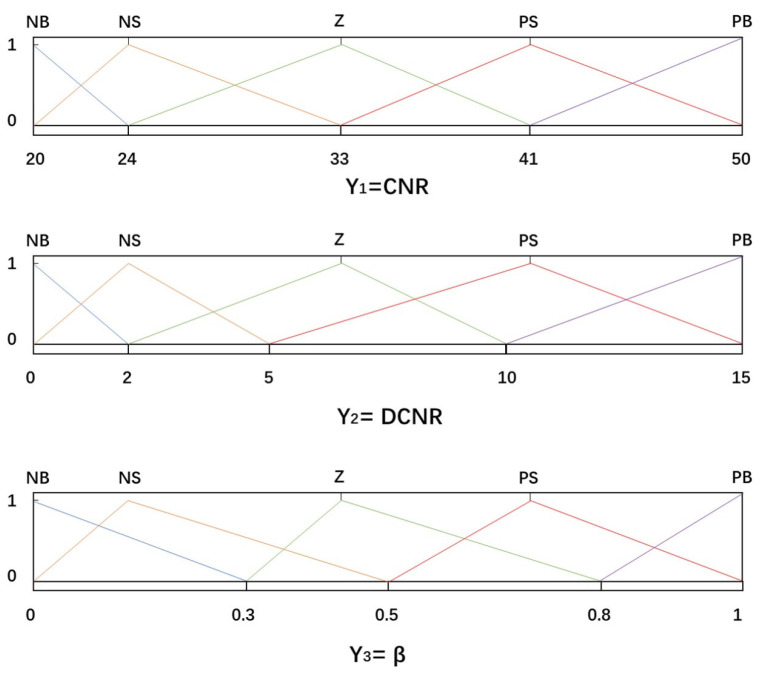
Membership function.

**Figure 6:**
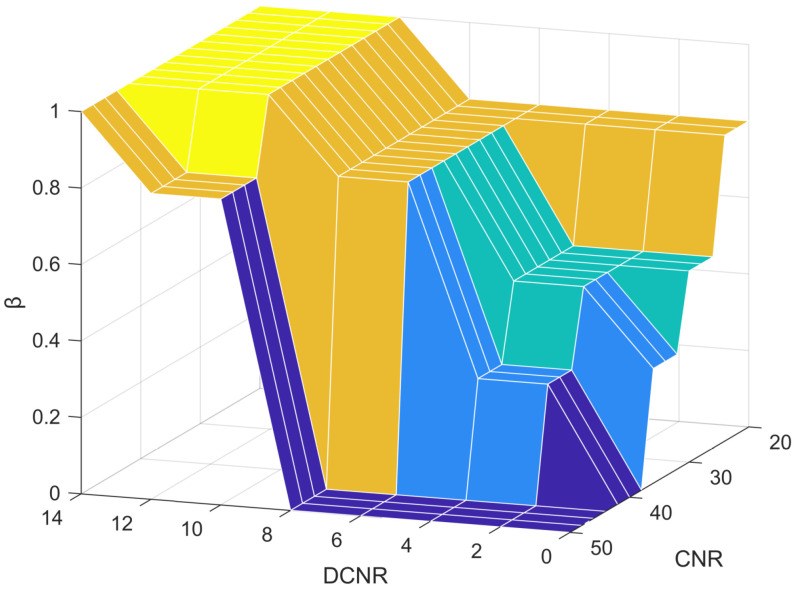
Fuzzy control rule.

As shown in Figure 5, the fuzzy controller adopts the reasoning mechanism of the Mamdani algorithm [27,28]. The Mamdani algorithm is a common algorithm in fuzzy control, which is used to map fuzzy input variables to fuzzy output variables in fuzzy control, and the language variables of input and output can be expressed as follows: Negative Big (NB), Negative Small (NS), Zero (Z), Positive Small (PS), and Positive Big (PB). The control rules can be expressed as $F\left[\langle Y_{1}=B_{1}^{j}\rangle \;\mathrm{and}\;\langle Y_{2}=B_{2}^{j}\rangle \right]\;\mathrm{Then}\;\left[Y_{3}=B_{3}^{j}\right]j=1,2,\ldots ,25$. Fuzzy control rules are mainly set according to the following principles: (1) switch to single-frequency mode when the CNR is unstable. (2) In the case of low CNR and instability, try to rely on the original position signal; in the case of stability, use dual-frequency working mode. (3) When the signal quality is improved, increase the connection between the two signals. The final specific rule formulation and adjustment is based on the actual simulation test, as shown in Figure 6.

Finally, the fuzzy output results calculated based on the Mamdani algorithm are defuzzified to obtain the final value. In this paper, the center of gravity method is adopted to defuzzify [29], namely,

(12) $$\begin{array}{c} \beta =\frac{\sum_{j}^{n_{j}}\beta_{j}\mu \left(\beta_{j}\right)}{\sum_{j}^{n_{j}}\mu \left(\beta_{j}\right)} \end{array}$$

For β values above 0.8, the ADCLS Processing Unit makes the loop work in single-frequency channel loops, and for β values below 0.8, the loop works in dual-frequency combined channel loops, and specifically changes the weight parameters of the code loop according to the value of β. The code loop phase discriminators obtained from the upper and lower side bands all adopt the incoherent advance and hysteresis power method, whose normalized phase discriminator formula is the following [30]:

(13) $$e=\frac{1}{2}\frac{E^{2}-L^{2}}{E^{2}+L^{2}}$$

In Equation (13), E and L represent the incoherent integration values, where E denotes the phase discriminator output. These values are combined and subsequently filtered by the tracking loop. The Numerically Controlled Oscillator (NCO) for both sidebands then generates the corresponding Weil codes. When the incoherent early-late amplitude method is applied, the code phase error can be expressed as a linear combination of the errors from the two sidebands. This results in a joint code loop discriminator output, which improves tracking accuracy.

(14) $$\begin{array}{c} disc\left(\tau \right)\;=\;\frac{\beta }{2}\frac{I{E_{o}}^{2}\;+\;Q{E_{o}}^{2}\;-\;I{L_{o}}^{2}\;-\;Q{L_{o}}^{2}}{I{E_{o}}^{2}\;+\;Q{E_{o}}^{2}\;+\;I{L_{o}}^{2}\;+\;Q{L_{o}}^{2}}\;+\;\frac{1\;-\;\beta }{2}\frac{I{E_{e}}^{2}\;+\;Q{E_{e}}^{2}\;-\;I{L_{e}}^{2}\;-\;Q{L_{e}}^{2}}{I{E_{e}}^{2}\;+\;Q{E_{e}}^{2}\;+\;I{L_{e}}^{2}\;+\;Q{L_{e}}^{2}} \end{array}$$

In the case of stable loop tracking, for carrier ring analysis, Equation (10) can be deformed to obtain the following expressions:

(15) $$\begin{array}{cc} \; & \frac{A_{e}}{A_{o}}QP_{o}\left(k\right)-QP_{e}\left(k\right)\\ \; & \approx \;2A_{e}T_{coh}\cos\left(\pi \Delta f_{c}T_{coh}\;+\;\Delta \theta_{c}\right)\sin\left(\pi \Delta f_{sc}T_{coh}\;+\;\Delta \theta_{sc}\right)\;\times \frac{A_{e}}{A_{o}}QP_{o}\left(k\right)+QP_{e}\left(k\right)\\ \; & \approx 2A_{e}T_{coh}\sin\left(\pi \Delta f_{c}T_{coh}\;+\;\Delta \theta_{c}\right)\cos\left(\pi \Delta f_{sc}T_{coh}\;+\;\Delta \theta_{sc}\right)quad\times \frac{A_{e}}{A_{o}}IP_{o}\left(k\right)+IP_{e}\left(k\right)\\ \; & \approx \;2\;A_{e}T_{\mathrm{coh}}\cos\left(\pi \Delta f_{c}T_{\mathrm{coh}}\;+\;\Delta \theta_{c}\right)\cos\left(\pi \Delta f_{\mathrm{sc}}T_{\mathrm{coh}}\;+\;\Delta \theta_{\mathrm{sc}}\right) \end{array}$$

As shown in the equation above, the primary carrier and subcarrier have been separated, allowing for the use of a two-quadrant arctangent function as the phase discriminator. Among various types of Costa Phase-Locked Loop (PLL) phase discriminators, the two-quadrant arctangent method provides the highest accuracy; however, it comes at the cost of increased computational complexity. The phase error can be derived from Equation (15) as follows:

(16) $$\begin{array}{cc} \mathrm{disc}\left(\theta_{sc}\right) & =\arctan\left(\frac{\frac{A_{e}}{A_{o}}QP_{o}\left(k\right)-QP_{e}\left(k\right)}{\frac{A_{e}}{A_{o}}IP_{o}\left(k\right)+IP_{e}\left(k\right)}\right)\approx \pi \Delta f_{sc}\;T_{\mathrm{coh}}+\Delta \theta_{sc}\\ \mathrm{disc}\left(\theta_{sc}\right) & =\arctan\left(\frac{\frac{A_{e}}{A_{o}}QP_{o}\left(k\right)+QP_{e}\left(k\right)}{\frac{A_{e}}{A_{o}}IP_{o}\left(k\right)+IP_{e}\left(k\right)}\right)\approx \pi \Delta f_{sc}\;T_{\mathrm{coh}}+\Delta \theta_{sc} \end{array}$$

After completing joint code loop tracking and carrier (both main and subcarrier) acquisition, the signal proceeds to the loop filtering stage. In this study, a second-order code loop is employed for code tracking. For carrier tracking, the main carrier loop initially uses a second-order Frequency-Locked Loop (FLL) to assist in frequency acquisition, followed by a third-order PLL for precise phase tracking. The subcarrier loop utilizes a second-order PLL. During the initial 350 ms of single-frequency tracking, the main carrier loop operates in FLL mode to pull into lock. Once frequency lock is achieved, the system transitions to the third-order PLL. Since the loop filters used in this paper are fundamentally similar to conventional designs, their detailed discussion is omitted.

### 2.3. Error Analysis

Prior to this section, the ADCLS signal, its propagation path, and the corresponding receiving and tracking loop have been described in detail. This section focuses on analyzing the ranging performance of the ADCLS system. The primary sources of measurement error in both the code loop and the Phase-Locked Loop include phase jitter and dynamic stress error. Phase jitter can be attributed to factors such as thermal noise, oscillator frequency jitter induced by mechanical vibrations, and Allan variance. Since the relative motion between the receiver and the base station is minimal during the testing process, dynamic stress errors can be neglected.

The primary factors affecting positioning accuracy in GPS receivers include thermal noise, mechanical vibrations, and Allan variance. This paper focuses on analyzing the impact of thermal noise, as it represents the dominant error source in both code and carrier tracking loops. Although mechanical vibrations can also degrade accuracy, their effects mainly appear as instantaneous phase jitter in carrier and pseudocode signals, caused by oscillator frequency modulation induced by mechanical disturbances. However, compared to thermal noise, their impact is relatively minor and is more dependent on the receiver’s motion state than on the intrinsic characteristics of the signal. Allan variance, which quantifies the frequency stability of the oscillator, is primarily determined by hardware specifications and becomes more significant under high-dynamic conditions. Since this study emphasizes the influence of thermal noise, a detailed discussion of Allan variance is omitted.

In laboratory tests, thermal noise is the primary source of error. Its impact on positioning accuracy is mainly reflected in the code loop, subcarrier, and main carrier tracking performance. As shown in Figure 1, the correlation between the two carriers is minimal, and the mutual influence can therefore be considered negligible. The mean square error due to thermal noise in the joint code loop can be expressed by the following equation:

(17) $$\begin{array}{ccc} \; & \sigma_{tdll}^{2}\approx \frac{B_{L}}{4\pi^{2}CNR}\; & \times \frac{\int_{-\frac{B_{fe}}{2}}^{\frac{B_{fe}}{2}}\left(p_{e}G_{e}\left(f\right){\left(1-\beta \right)}^{2}\sin^{2}\left(\frac{\pi fT_{\mathrm{coh}\;}}{2}\right)+p_{o}G_{o}\left(f\right)\beta^{2}\sin^{2}\left(\frac{\pi fT_{\mathrm{coh}\;}}{2}\right)\right)df}{{\left(\int_{-\frac{B_{fe}}{2}}^{\frac{B_{fe}}{2}}\left(p_{e}G_{e}\left(f\right){\left(1-\beta \right)}^{2}\sin\left(\frac{\pi fT_{\mathrm{coh}\;}}{2}\right)+p_{o}G_{o}\left(f\right)\beta^{2}\sin\left(\frac{\pi fT_{\mathrm{coh}\;}}{2}\right)\right)df\right)}^{2}} \end{array}$$

(18) $$\begin{array}{ccc} \; & \sigma_{cdll}^{2}=\sigma_{tdll}^{2}\times \left(1+\frac{B_{L}}{T_{coh}CNR}\right.\; & \times \frac{\int_{-\frac{B_{fe}}{2}}^{\frac{B_{fe}}{2}}\left(p_{e}G_{e}\left(f\right){\left(1-\beta \right)}^{2}\cos^{2}\left(\frac{\pi fT_{coh}}{2}\right)+p_{o}G_{o}\left(f\right)\beta^{2}\cos^{2}\left(\frac{\pi fT_{coh}}{2}\right)\right)df}{{\left(\int_{-\frac{B_{fe}}{2}}^{\frac{B_{fe}}{2}}\left(p_{e}G_{e}\left(f\right){\left(1-\beta \right)}^{2}\cos\left(\frac{\pi fT_{coh}}{2}\right)+p_{o}G_{o}\left(f\right)\beta^{2}\cos\left(\frac{\pi fT_{coh}}{2}\right)\right)df\right)}^{2}} \end{array}$$

In Equation (17), $B_{fe}$ is the RF front-end bandwidth, D is the correlates distance, CNR is the carrier-to-noise ratio, the correlate distance is set to 0.5, and the thermal noise variance of the joint code ring after calculating the square loss is Equation (18) [31].

The thermal noise variance of the main carrier loop and the subcarrier loop is shown in the formula below:

(19) $$\begin{array}{c} \sigma_{cpll}^{2}\approx \frac{B_{CPLL}}{NR\int_{-\frac{B_{fe}}{2}}^{\frac{B_{fe}}{2}}\left(p_{e}G_{e}\left(f\right)+p_{o}G_{o}\left(f\right)\right)df}\\ \sigma_{scll}^{2}\approx \frac{B_{SCPLL}}{NR\int_{-\frac{B_{fe}}{2}}^{\frac{B_{fe}}{2}}\left(p_{e}G_{e}\left(f\right)+p_{o}G_{o}\left(f\right)\right)df} \end{array}$$

In Equation (19), $B_{CPLL}$ and $B_{SCPLL}$ represent the noise bandwidth of the main carrier loop and the subcarrier loop, respectively, and the mean square error of thermal noise is the main source affecting the positioning accuracy.

## 3. Simulation Results

### Experimental Conditions

In the experiment, the N5182A MXG vector signal generator from Keysight (Santa Rosa, CA, USA) was used as the signal source. It supports frequencies ranging from 100 kHz to 6 GHz and is capable of generating multi-channel baseband and RF signals. For ADCLS signal generation, the carrier frequency of the original positioning signal was set to 3500 MHz, using CNFS positioning code group 1 (PRN1) with a code rate of 10.23 MHz and a bandwidth of 20.46 MHz. The embedded positioning signal was configured at 3494.39 MHz, using code group 2 (PRN5) with a code rate of 1.023 MHz and a bandwidth of 2.046 MHz. The communication and positioning signals were transmitted in a fused manner, and additive white Gaussian noise was introduced during signal generation. At the receiving end, the Tapped Delay Line (TDL-D) channel model was implemented using simulation software. This model, commonly used in 5G NR link-level evaluations, captures the multipath delay and Doppler effects characterizing time-varying channels [32,33]. The receiver hardware was a USRP B210 device from Caisa Technology (Shenzhen, China), with data acquisition performed via recplay. The collected data were processed in the software terminal, where the 5G link model was configured. Since the CNFS transmits both communication and navigation signals through a shared antenna, the 5G TDL model is well suited for its evaluation. The received CNFS signals are composed of multiple time-delayed multipath components. Due to the unique characteristics of the ADCLS signal, this study compares the proposed method against the most widely used joint tracking loop algorithm currently available.

**Figure 7:**
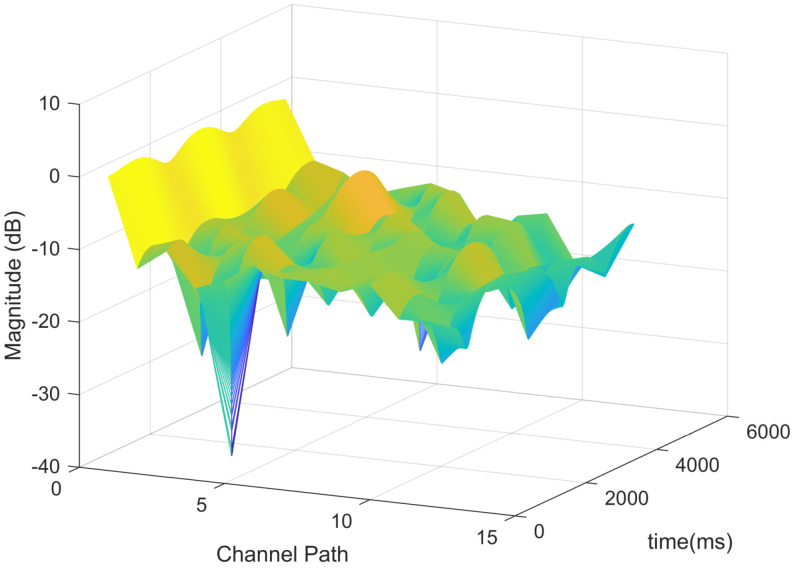
Time-varying fading characteristics of TDL-D.

The TDL model was originally designed for Single-Input Single-Output (SISO) systems, covering a frequency range from 0.5 GHz to 100 GHz with a maximum supported bandwidth of 2 GHz. In this study, the TDL-D variant is employed, with its specific configuration parameters detailed in Table 1. Each tap’s Doppler spectrum in the TDL-D model follows the classical Jakes spectrum, characterized by a maximum Doppler frequency shift FD. Notably, the first tap in the TDL-D model experiences Rician fading, while subsequent taps typically exhibit Rayleigh fading behavior. In this setup, the maximum Doppler shift is set to 200 Hz. Figure 7 illustrates the time-varying fading characteristics modeled by the TDL-D channel.

The capture and tracking analysis was carried out on a PC, and the simulation software was run on the PC. The configuration of the PC was I5-1135G7, with 32 G running memory and 2 TB solid-state memory. The terminal analog software can track up to eight separate signals at the same time or carry out four joint tracking operations at the same time. The specific parameters received by the terminal are shown in the table.

The signal acquisition and tracking analysis were conducted on a personal computer (PC) running the simulation software. The PC was configured with an Intel Core i5-1135G7 processor, 32 GB of RAM, and a 2 TB solid-state drive (Intel, Santa Clara, CA, USA). The terminal simulation software supports simultaneous tracking of up to eight individual signals or four joint-tracking channels. The detailed configuration parameters of the received signals at the terminal are summarized in Table 1.

**Figure 8:**
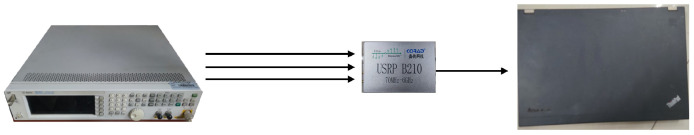
Schematic diagram of laboratory equipment connection.

We connected the signal source generators B210 and PC, respectively, as shown in Figure 8.

**Figure 9:**
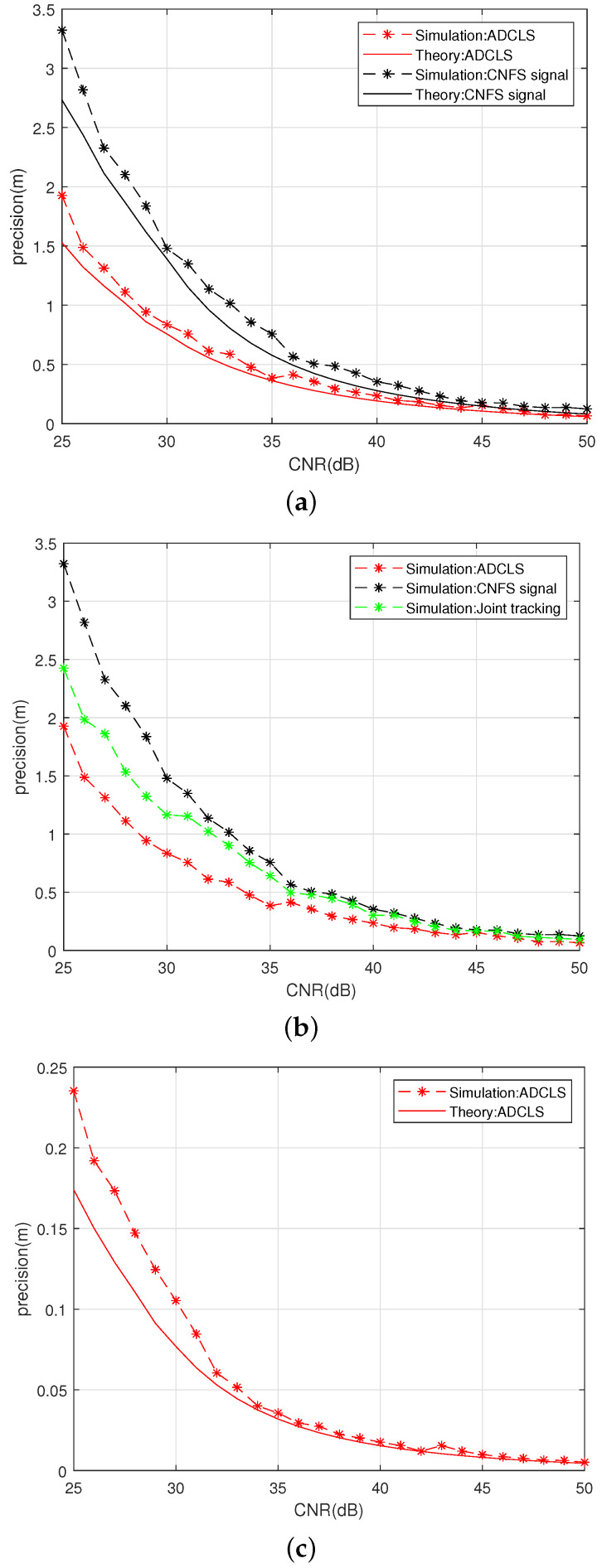
Thermalnoise error theory and experimental test diagram: (**a**) Code loop noise error. (**b**) Main carrier noise error. (**c**) Subcarrier loop noise error.

Using the connection method of Figure 8 ADCLS signal source, we use a fuzzy control-based asymmetric ring joint double-carrier tracking loop for reception to test the tracking precision of the code loop, carrier loop, and subcarrier loop under different CNR conditions for the ADCLS signal receiving loop based on fuzzy control asymmetric ring joint double-carrier tracking, as shown in Figure 9.

Figure 9 illustrates that the experimental results closely align with the theoretical calculations. Under experimental conditions, the tracking accuracy of the ADCLS signal—using the fuzzy control-based asymmetric dual-carrier joint reception and tracking loop—shows an average improvement of 44.08% compared with the traditional channel fusion positioning signal under low carrier-to-noise ratio (CNR) conditions. Specifically, the carrier loop accuracy is enhanced by 33.66%. This improvement is attributed to the utilization of both the original and embedded signals, which share the same source and exhibit strong channel correlation. The loop design effectively combines the phase discrimination information from the upper and lower sidebands, thereby enhancing the stability and accuracy of the code loop tracking. Under high=CNR conditions, the code loop tracking accuracy improves by an average of 32.76%, while the carrier loop tracking accuracy improves by 40.54%. These gains are mainly due to the asymmetric phase discrimination strategy enabled by fuzzy control.

Overall, the incorporation of an additional subcarrier into the ADCLS signal superimposed on the original signa leads to a substantial enhancement in the tracking accuracy of the subcarrier loop. Due to the broader effective bandwidth of the Gabor-based ADCLS, the subcarrier loop achieves an accuracy approximately an order of magnitude greater than that of the code loop. This architectural design allows the system to fully leverage both frequency components, thereby enabling more reliable and precise single-point positioning.

**Figure 10:**
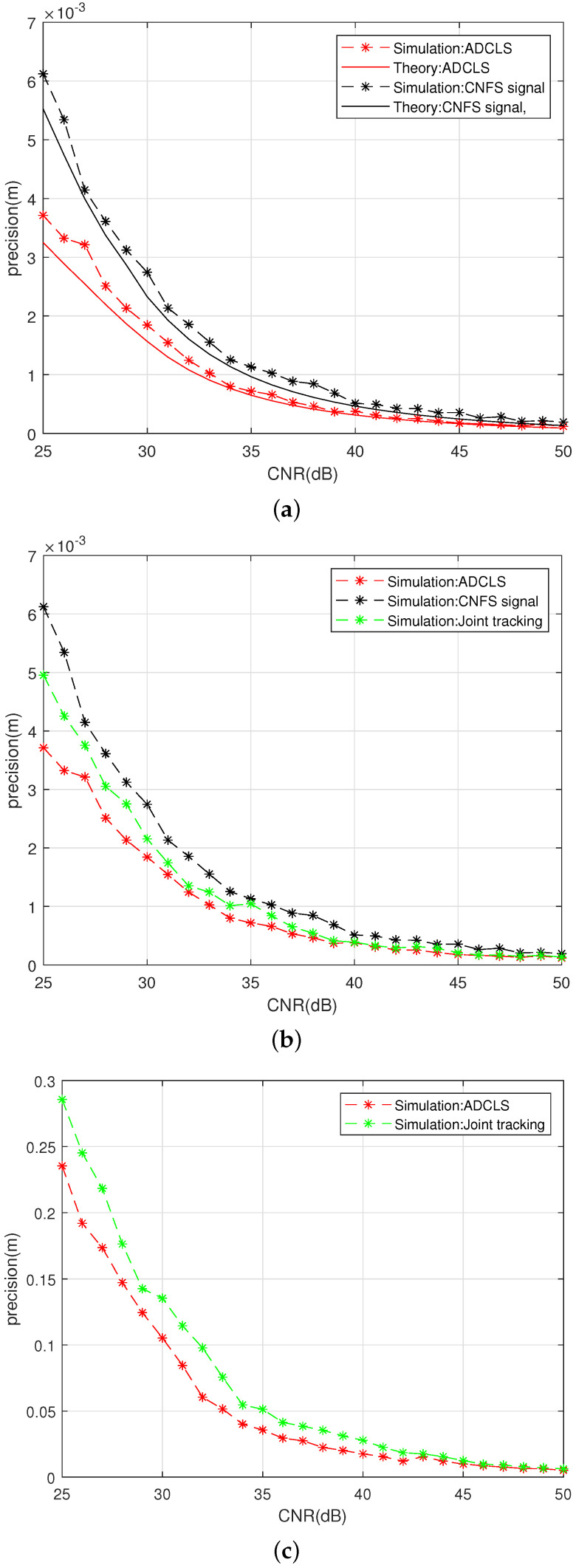
Comparison diagram of different tracking loops: (**a**) Code loop noise error. (**b**) Main carrier noise error. (**c**) Subcarrier loop noise error.

To evaluate the tracking performance of the proposed loop in the ADCLS system, a comparative analysis was conducted between the traditional joint tracking algorithm and the dual-carrier dual-code loop with fuzzy control introduced in this study. Both methods were tested under simultaneous tracking conditions for ADCLS signals. The comparative results are presented in Figure 10.

Figure 10 presents a comparison of the tracking accuracy for the code loop, primary carrier loop, and subcarrier loop between the conventional joint tracking algorithm and the proposed fuzzy control-based dual-carrier dual-code tracking algorithm. The evaluation was conducted under both simultaneous tracking of ADCLSs and single-channel tracking of the original positioning signal. Under low-carrier-to-noise-ratio (CNR) conditions, the proposed algorithm achieves improvements of 38.55% in code loop tracking accuracy, 19.16% in primary carrier loop accuracy, and 22.39% in subcarrier loop accuracy compared to the conventional joint tracking algorithm. These results demonstrate that the fuzzy control mechanism in the proposed method effectively leverages signal channel correlation and integrates upper and lower sideband phase discriminators to enhance tracking performance. Overall, the proposed approach offers superior tracking precision under challenging signal conditions.

## 4. Conclusions

This paper primarily focuses on three key technical advancements within integrated communication and navigation systems. Firstly, at the signal design level, an embedded signal is introduced and reconfigured into an asymmetric double-sideband positioning signal. By leveraging the common signal source and channel correlation between the two signals, this design enhances the Gabor bandwidth, thereby theoretically improving the upper limit of signal positioning capability. Secondly, at the receiver loop level, a fuzzy-control-based asymmetric dual-carrier loop joint reception tracking architecture is developed specifically for the new signal structure. This approach optimally exploits both signal correlations and Gabor bandwidth characteristics, achieving enhanced loop tracking accuracy and ultimately superior ranging precision. Thirdly, signal generation and simulation-based testing validate the proposed methodology, demonstrating the technical superiority of the integrated signal design and receiver loop system through comprehensive performance evaluations.

## Figures and Tables

**Table 1:** Experimental parameter setting.

Parameter	Parameter Settings
Coherent integration time	1 ms
Sampling frequency	50 MHz
Code ring noise bandwidth	1 Hz
Main carrier noise bandwidth	10 Hz
Subcarrier noise bandwidth	0.8 Hz
Incoherent integration time	1 ms
Correlator spacing	0.5
Sampling bit	8 bit
Damping coefficient	0.707
Traction time	350 ms
PMF-FFT numbers	128

## Data Availability

The data supporting the findings of this study are available from the corresponding author upon reasonable request.
